# Inhibitory Effects of Extracellular Vesicles from iPS-Cell-Derived Mesenchymal Stem Cells on the Onset of Sialadenitis in Sjögren’s Syndrome Are Mediated by Immunomodulatory Splenocytes and Improved by Inhibiting miR-125b

**DOI:** 10.3390/ijms24065258

**Published:** 2023-03-09

**Authors:** Qingguo Zhao, Eun-Hye Bae, Yu Zhang, Arash Shahsavari, Pranayvir Lotey, Ryang Hwa Lee, Fei Liu

**Affiliations:** Department of Cell Biology and Genetics, School of Medicine, Texas A&M University Health Science Center, College Station, TX 77843, USA

**Keywords:** extracellular vesicles, mesenchymal stem cells, iPS cells, Sjögren’s syndrome, miR-125b, splenic macrophages

## Abstract

Extracellular vesicles (EVs) from allogeneic-tissue-derived mesenchymal stem cells (MSCs) are promising to improve Sjögren’s syndrome (SS) treatment, but their application is hindered by high variations in and limited expandability of tissue MSCs. We derived standardized and scalable MSCs from iPS cells (iMSCs) and reported that EVs from young but not aging iMSCs (iEVs) inhibited sialadenitis onset in SS mouse models. Here, we aim to determine cellular mechanisms and optimization approaches of SS-inhibitory effects of iEVs. In NOD.B10.H2^b^ mice at the pre-disease stage of SS, we examined the biodistribution and recipient cells of iEVs with imaging, flow cytometry, and qRT-PCR. Intravenously infused iEVs accumulated in the spleen but not salivary glands or cervical lymph nodes and were mainly taken up by macrophages. In the spleen, young but not aging iEVs increased M2 macrophages, decreased Th17 cells, and changed expression of related immunomodulatory molecules. Loading miR-125b inhibitors into aging iEVs significantly improved their effects on repressing sialadenitis onset and regulating immunomodulatory splenocytes. These data indicated that young but not aging iEVs suppress SS onset by regulating immunomodulatory splenocytes, and inhibiting miR-125b in aging iEVs restores such effects, which is promising to maximize production of effective iEVs from highly expanded iMSCs for future clinical application.

## 1. Introduction

Sjögren’s syndrome (SS), a chronic inflammatory autoimmune disease, affects mainly salivary glands and lacrimal glands [1,2]. The consequent long-term dry mouth (xerostomia) exacerbates dental caries and periodontal disease and causes problems of taste, sleep, and speech, which severely impair quality of life. No therapy for SS has demonstrated to be really effective, and current therapeutic management is still based on the symptomatic treatment of sicca symptomatology and a variety of immunosuppressive agents for systemic features [3].

Mesenchymal stem cells (MSCs), multipotent stem cells isolated from bone marrow or various other tissues, can promote regeneration and modulate immune responses mainly through paracrine effects [4]. In preclinical studies and a few small clinical trials, allogeneic- but not autologous-tissue-derived MSCs alleviated xerostomia caused by SS after systemic infusion [5,6]. However, the clinical application of tissue-derived MSCs is hindered by their high functional variations (due to differences in donors and source tissues, methods of isolation and expansion), limited expandability, loss of therapeutic activities after prolonged expansion, safety concerns associated with live cell treatment, dynamic changes in vitro and in vivo, and high cost and infrastructure requirements [7,8,9].

To overcome limitations of tissue-derived MSCs, we derived standardized MSCs efficiently from transgene-free human induced pluripotent stem cells (iPSCs) with a theoretically limitless expandability [10]. The anti-inflammatory and pro-regenerative properties of our iPSC-derived MSCs (iMSCs) are superior or comparable to the best batches of bone marrow MSCs tested in our NIH-funded MSC distribution center and are consistent between different derivation batches [11,12,13]. Extracellular vesicles (EVs), including exosomes and microvesicles, carry bioactive molecules from their parent cells and facilitate the delivery of these molecules into recipient cells. EVs from tissue-derived MSCs show anti-inflammatory and pro-regenerative properties similar to MSCs and appear more feasible for clinical applications than live cells, but their application is still hindered by variations and the limited expandability of source MSCs [14,15]. Recently, we found that when infused intravenously at the pre-disease stage, EVs from early-passage iMSCs (young iEVs) but not from late-passage iMSCs (aging iEVs) can inhibit the onset of sialadenitis with efficiency comparable to young iMSCs and bone marrow MSCs in mouse SS models [12,16]. The loss of SS-inhibitory effects in aging iEVs is related to the decreased TGFβ1 and miR-21 and increased miR-125b levels compared with young iEVs [16]. Here, we report that splenic macrophages are a major type of recipient cells of intravenously infused iEVs, and young iEVs but not aging iEVs inhibit the onset of SS, likely through increasing M2 macrophages and decreasing T helper 17 (Th17) cells in the spleen, whereas inhibiting miR-125b in aging iMSCs make their EVs as competent as young iEVs in these activities.

## 2. Results

### 2.1. Systemically Infused iEVs Were Mainly Taken up by Macrophages in Spleen

**Figure 1:**
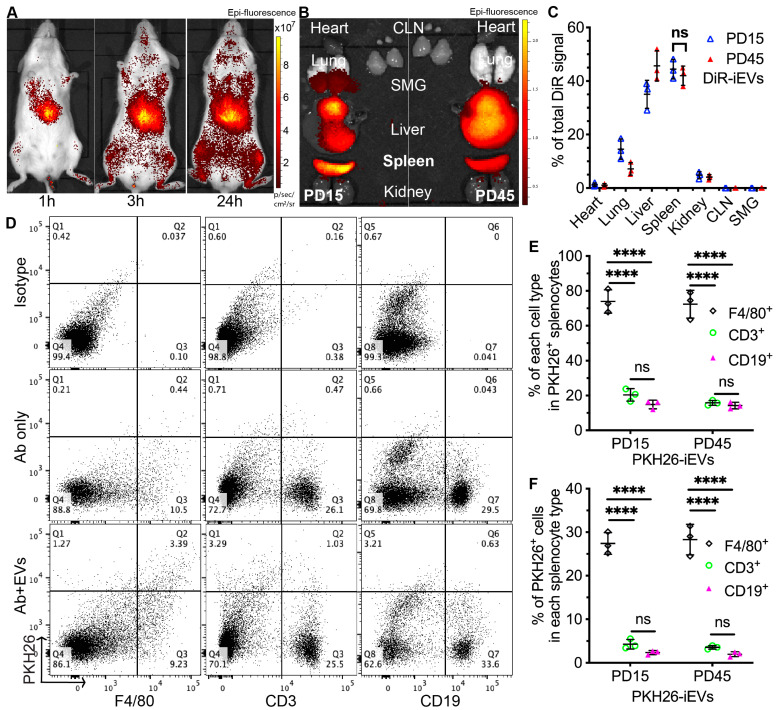
The biodistribution of intravenously infused iEVs. (**A**–**C**) Four-month-old female NOD.B10.H2^b^ mice were IV injected with PD15 and PD45 iEVs (1.5 × 10^10^ EVs per mouse) labeled with DiR. The DiR signal in live mice was imaged at 1, 3, and 24 h after injection (p/s/cm^2^/sr: photon counts per second per centimeter squared per steradian) (**A**). Major organs were collected at 24 h after injection for ex vivo DiR imaging and quantification of relative DiR epi-fluorescence intensities (**B**,**C**). N = 3, ns: not significant, *p* > 0.05. (**D**–**F**) Splenocytes were isolated from 4-month-old female NOD.B10.H2^b^ mice and cultured with PD15 and PD45 iEVs (1 × 10^9^ EVs/mL) labeled with PKH26 for 3 h. After washing with PBS, types of PKH26^+^ splenocytes were examined with flow cytometry and quantified. N = 3, ****: *p* < 0.0001. All quantitative data are shown as individual values with Mean ± SD.

To trace the biodistribution of iEVs in NOD.B10.H2^b^ mice, a model of primary SS, PD15 iEVs (population doubling 15; early-passage iMSC-derived EVs; young iEVs), and PD45 iEVs (late-passage iMSC-derived EVs; aging iEVs) were labeled with a near-infrared fluorescent dye, DiR, as reported in [17], and IV infused into 4-month-old female NOD.B10.H2^b^ mice. At 1, 3, and 24 h after injection, mice were imaged in vivo, and then major organs were collected for ex vivo DiR imaging. Imaging data indicated that DiR signals were mainly present in the upper abdomen regions, strongest in the liver and spleen, but not detected in submandibular glands (SMGs) or cervical lymph nodes (CLNs) collected at 24 h after injection of either young or aging iEVs (Figure 1A–C). Since autoreactive cells in the spleen drive disease manifestations of SS [18], the above data suggest that iEVs might suppress the autoimmune responses in NOD.B10.H2^b^ mice by modulating splenocytes. To determine which types of cells in the spleen take up iEVs, we labeled both young and aging iEVs with a fluorescent dye, PKH26, as in reported [19], isolated splenocytes from 4-month-old female NOD.B10.H2^b^ mice, and treated them with PKH26-labeled EVs for 3 h. These splenocytes were then subjected to flow cytometry analysis for PKH26 and markers for macrophages (F4/80), T cells (CD3), or B cells (CD19). In both young and aging iEV groups, most PKH26^+^ splenocytes were F4/80^+^ (>70%), whereas much fewer PKH26^+^ splenocytes were CD3^+^ or CD19^+^ (Figure 1D,E). In these different types of splenocytes, the percentage of PKH26^+^ was also significantly higher in F4/80^+^ cells than in CD3^+^ or CD19^+^ cells (Figure 1F). These data indicated that macrophages are the major population uptaking iEVs in the spleen of NOD.B10. H2^b^ mice.

### 2.2. Young iEVs but Not Aging iEVs Promoted M2 Polarization of Splenic Macrophages

**Figure 2:**
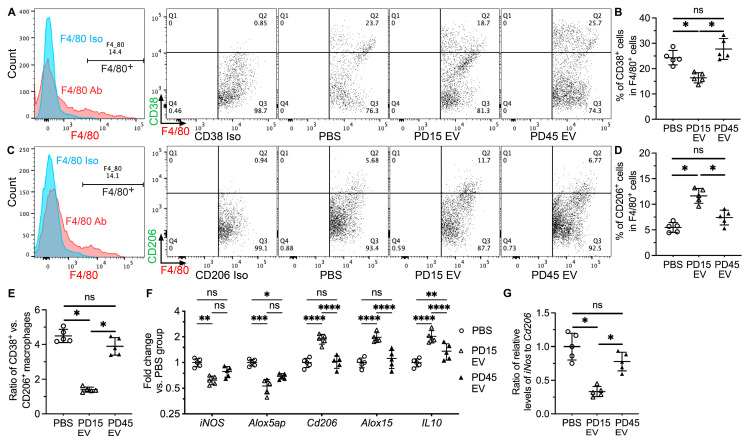
Effects of iEVs on the polarization of splenic macrophages. Four-month-old female NOD.B10.H2^b^ mice were IV injected with PBS, PD15 iEVs, or PD45 iEVs twice a week for two weeks. Splenocytes were collected at two weeks after last injection. (**A**–**E**) Percentages of CD38^+^ or CD206^+^ cells in F4/80^+^ splenocytes were examined with flow cytometry, and the ratio of CD38^+^ vs. CD206^+^ cells in F4/80^+^ splenocytes was calculated. (**F**,**G**) The mRNA levels of markers for M1 or M2 macrophages in splenocytes were examined with qRT-PCR, and the ratio of relative levels of iNos to Cd206 was calculated. N = 5, ns: not significant, *: *p* < 0.05, **: *p* < 0.01, ***: *p* < 0.001, ****: *p* < 0.0001.

The pathogenesis of SS involves the polarization of macrophages into the pro-inflammatory M1 phenotype [20], whereas EVs from tissue-derived MSCs reprogram macrophages into the anti-inflammatory M2 phenotype in mouse models of several inflammation-related diseases [21,22]. Since the spleen is essential for the pathogenesis of SS [18] and the primary target organ of IV infused iEVs, we examined the polarization of splenic macrophages from NOD.B10.H2^b^ mice at 2 weeks after last iEV treatment with flow cytometry. In F4/80^+^ splenic macrophages, IV infusion of PD15 iEVs but not PD45 iEVs significantly decreased percentages of CD38^+^ M1 macrophages, increased percentages of CD206^+^ M2 macrophages, and decreased the ratio of CD38^+^ vs. CD206^+^ cells (Figure 2A–E). Consistently, qRT-PCR assays indicated that compared with the PBS group, IV infusion of PD15 iEVs significantly decreased the mRNA expression of M1 macrophage markers *iNos* and *Alox5ap* and increased M2 macrophage markers *Cd206*, *Alox15*, and *IL10* in the spleen, whereas PD45 iEVs only significantly affected *Alox5ap* and *IL10* expression among these markers to a lesser extent (Figure 2F). Moreover, only PD15 but not PD45 iEVs significantly decreased the ratio of relative mRNA levels of *iNos* to *Cd206* (Figure 2G). These data indicate that young but not aging iEVs shifted the polarization of splenic macrophages to the anti-inflammatory M2 phenotype.

### 2.3. Young iEVs but Not Aging iEVs Decreased Th17 Cells in Spleen

**Figure 3:**
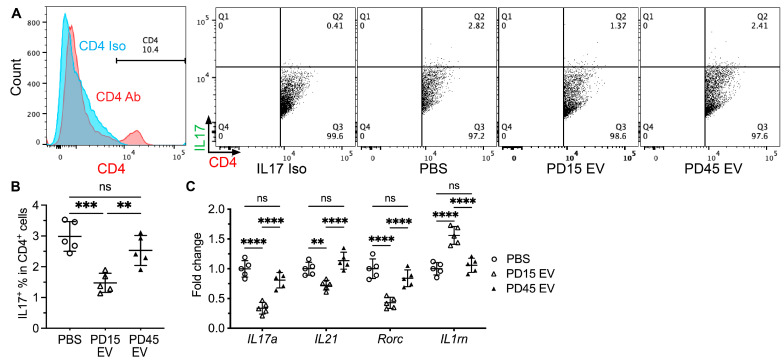
Effects of iEVs on splenic Th17 cells. Four-month-old female NOD.B10.H2^b^ mice were IV injected with PBS, PD15 iEVs, or PD45 iEVs twice a week for two weeks. Splenocytes were collected at two weeks after last injection. (**A**,**B**) Percentages of IL17^+^ cells in splenic CD4^+^ T cells were examined with flow cytometry. (**C**) The mRNA levels of Th17 markers (*IL17a*, *IL21*, and *Rorc*) and negative regulator *IL1rn* in spleen were examined with qRT-PCR. N = 5, ns: not significant, **: *p* < 0.01, ***: *p* < 0.001, ****: *p* < 0.0001.

T helper 17 (Th17) cells are important drivers of SS in various mouse models including the NOD.B10.H2^b^ mice [23,24,25], whereas EVs from tissue-derived MSCs suppressed development of Th17 cells in mouse models of several other autoimmune diseases [26]. In CD4^+^ spleen Th cells from NOD.B10.H2^b^ mice at 2 weeks after iEV treatment, as mentioned in Section 2.2, IV infusion of PD15 iEVs but not PD45 iEVs significantly decreased percentages of IL17^+^ cells (Figure 3A,B). Consistently, the qRT-PCR assay indicated that compared with the PBS group, PD15 iEVs but not PD45 iEVs significantly decreased the mRNA expression of Th17 markers including *IL17a*, *IL21*, and *Rorc* in the spleen (Figure 3C). Regulatory T (Treg) cells are also involved in the development of SS, whereas it is not the number but rather the function of Tregs that is the driving factor, as indicated in the NOD.B10.H2^b^ mouse model [25]. In spleens of NOD.B10.H2^b^ mice after iEV treatment, neither PD15 nor PD45 iEVs significantly affected percentages of Foxp3^+^ cells in splenic CD4^+^ T cells or the mRNA levels of Treg markers *Foxp3*, *IL2ra/Cd25*, and *Tgfb1* (Supplementary Figure S1). Since IV-infused iEVs were predominantly taken up by macrophages in the spleen, the effects of these iEVs on Th17 differentiation are likely mediated by macrophages through IL1 signaling, as reported in [27]. Consistently, PD15 iEVs but not PD45 iEVs significantly increased the mRNA level of IL1 antagonist *IL1rn* in the spleen (Figure 3C). These data indicate that young iEVs but not aging iEVs inhibited Th17 differentiation in the spleen, likely through promoting M2 macrophage polarization.

### 2.4. Inhibition of miR-125b in Aging iMSCs Enabled Their EVs to Repress the Onset of Sialadenitis

**Figure 4:**
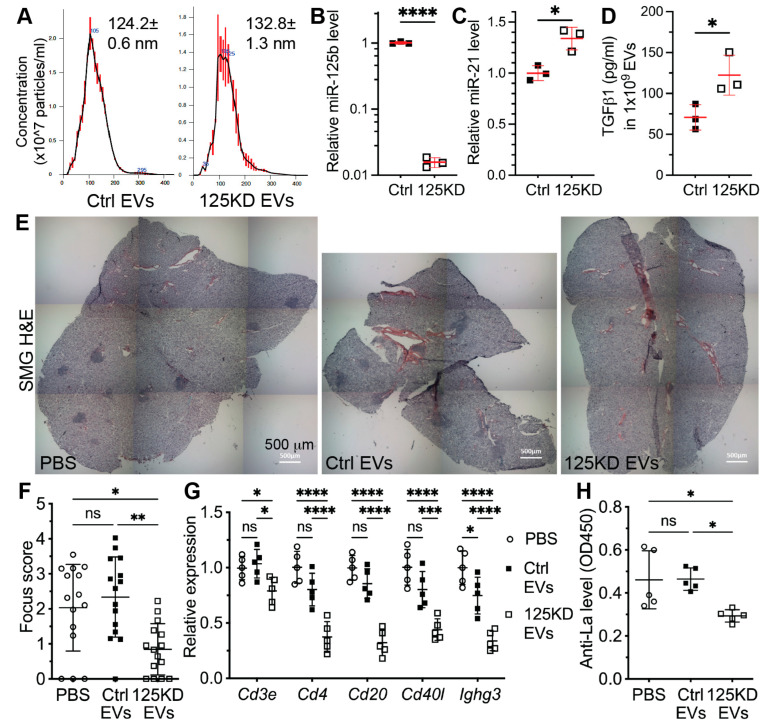
Effects of inhibiting miR-125b in aging iEVs on their in vivo capacity to repress the onset of sialadenitis. (**A**–**D**) Ctrl or 125KD EVs were isolated from PD45 iMSCs transfected with control or miR-125b inhibitors. Their mean sizes were measured with NanoSight nanoparticle tracking analysis (**A**). N = 3, EV concentrations at different sizes were shown as Mean (black) ± SEM (red). The levels of active miR-125b and miR-21 in these EVs were measured with qPCR (**B**,**C**). The level of TGFβ1 protein in these EVs was measured with ELISA (**D**). N = 3, Means and SDs are shown in red. (**E**–**H**) Four-month-old female NOD.B10.H2^b^ mice were IV injected with PBS, Ctrl EVs, or 125KD EVs twice a week for two weeks. At two weeks after last injection, SMGs were collected for H&E staining and representative images of whole SMG sections were shown by combining all fields taken at the lowest magnification of microscope (**E**). Focus scores were consequently quantified (**F**). These SMGs were also examined with qRT-PCR analyses of markers for T and B lymphocytes (**G**). The serum level of anti-La autoantibody was measured with ELISA (**H**). N = 5, ns: not significant, *: *p* < 0.05, **: *p* < 0.01, ***: *p* < 0.001, ****: *p* < 0.0001.

MicroRNAs are important mediators of the immune modulatory effects of MSC EVs [28]. We identified that miR-125b is highly enriched in aging iEVs compared with young iEVs, and the inhibition of miR-125b in aging iEVs increased their activities in suppressing Th17 responses in LPS-stimulated splenocytes [16]. Therefore, here we examined whether the inhibition of miR-125b can improve their inhibitory effects on SS onset in NOD.B10.H2^b^ mice. Aging iEVs were isolated from late-passage (PD45) iMSCs transfected with control or miR-125b inhibitors, as we reported in [16], and termed Ctrl EVs or 125KD (knockdown) EVs. The mean sizes of Ctrl EVs and 125KD EVs were comparable, whereas the level of miR-125b detectable by qPCR was remarkably lower in 125KD EVs, as expected (Figure 4A,B). Interestingly, levels of miR-21 and the TGFβ1 protein, two immune suppressive molecules enriched in young iEVs, were significantly higher in 125KD EVs than in Ctrl EVs (Figure 4C,D), indicating that the change in 125KD iEVs is not limited to the decrease in miR-125b activity. These modified aging iEVs or PBS were infused into female 4-month-old NOD.B10.H2^b^ mice at the pre-disease stage via IV twice a week for two weeks, as we reported in [16]. Two weeks after the last injection, we collected SMGs and serum samples for the following analyses. H&E staining of SMG sections indicated that the size of leukocyte infiltrates in SMGs significantly decreased by 125KD EVs but not Ctrl EVs compared with the PBS group (Figure 4E,F). Consistently, qRT-PCR assays indicated that the mRNA levels of markers for T cells (*Cd3* and *Cd4*), B cells (*Cd20* and *Ighg3*), and their activation (*Cd40l*) in SMGs were significantly decreased by 125KD EVs, whereas only one of these markers, *Ighg3*, was significantly downregulated by Ctrl EVs and the downregulation was much weaker than 125KD EVs (Figure 4G). The serum level of the anti-La autoantibody was also significantly decreased by 125KD EVs but not Ctrl EVs (Figure 4H). These data indicate that the transfection of miR-125b inhibitors into aging iMSCs can improve repressive effects of their EVs on the onset of sialadenitis in the NOD.B10.H2^b^ mouse model of primary SS.

### 2.5. Inhibition of miR-125b in Aging iEVs Restored Their Activity to Promote M2 Polarization

**Figure 5:**
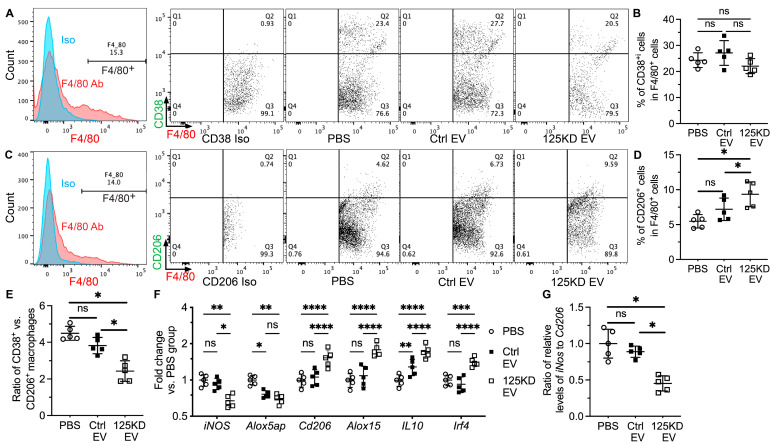
Effects of inhibiting miR-125b in aging iEVs on the polarization of splenic macrophages. Four-month-old female NOD.B10.H2^b^ mice were IV injected with PBS, Ctrl EVs, or 125KD EVs twice a week for two weeks. Splenocytes were collected at two weeks after last injection. (**A**–**E**) Percentages of CD38^+^ or CD206^+^ cells in F4/80^+^ macrophages were examined with flow cytometry, and the ratio of CD38^+^ vs. CD206^+^ cells in F4/80^+^ splenocytes was calculated. (**F**,**G**) The mRNA levels of markers for M1 or M2 macrophages in splenocytes were examined with qRT-PCR, and the ratio of relative levels of iNos to Cd206 was calculated. N = 5, ns: not significant, *: *p* < 0.05, **: *p* < 0.01, ***: *p* < 0.001, ****: *p* < 0.0001.

Previous studies showed that the overexpression of miR-125b induced the proinflammatory activation of macrophages and consequent T cell activation through inhibiting the expression of Irf4 [29,30]. Since our data above indicated that splenic macrophages are the major cells up taking iEVs and young iEVs but not aging iEVs promoted M2 polarization of splenic macrophages (Figure 1 and Figure 2), we further examined the effects of miR-125b inhibition in aging iEVs on the polarization of splenic macrophages in NOD.B10.H2^b^ mice at 2 weeks after IV infusion of Ctrl or 125KD aging iEVs. In F4/80^+^ splenic macrophages, 125KD but not Ctrl iEVs significantly decreased percentages of CD38^+^ M1 macrophages, increased percentages of CD206^+^ M2 macrophages, and decreased the ratio of CD38^+^ vs. CD206^+^ cells (Figure 5A–E). As indicated by qRT-PCR assay, the mRNA expression of Irf4, an miR-125b target essential for M2 macrophage polarization [29,31], was significantly increased by 125KD iEVs compared to either the PBS or Ctrl iEV groups (Figure 5F). Consistently, compared with the PBS group, 125KD iEVs significantly decreased the mRNA expression of M1 macrophage markers iNos and Alox5ap and increased M2 macrophage markers *Cd206*, *Alox15*, and *IL10* in the spleen, whereas Ctrl iEVs only significantly affected *Alox5ap* and *IL10* expression among these markers to lesser extent (Figure 5F). Moreover, only 125KD but not Ctrl iEVs significantly decreased the ratio of relative mRNA levels of *iNos* to *Cd206* (Figure 5G). These data indicate that the transfection of miR-125b inhibitors into aging iMSCs can restore the activity of their EVs to promote M2 polarization of splenic macrophages.

### 2.6. Inhibition of miR-125b in Aging iEVs Improved Their Activity to Decrease Splenic Th17 Cells

**Figure 6:**
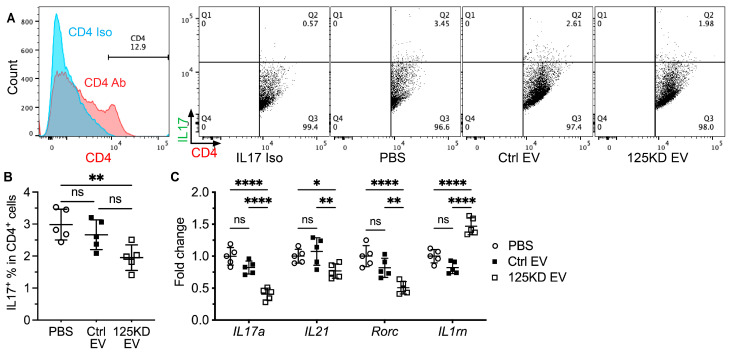
Effects of inhibiting miR-125b in aging iEVs on the polarization of splenic macrophages. Four-month-old female NOD.B10.H2^b^ mice were IV injected with PBS, Ctrl EVs, or 125KD EVs twice a week for two weeks. Splenocytes were collected at two weeks after last injection. (**A**,**B**) Percentages of IL17^+^ cells in CD4^+^ splenocytes were examined with flow cytometry. (**C**) The mRNA levels of Th17 markers (IL17a, IL21, and Rorc) and negative regulator IL1rn in spleen were examined with qRT-PCR. N = 5, ns: not significant, *: *p* < 0.05, **: *p* < 0.01, ****: *p* < 0.0001.

Our data above showed that young iEVs but not aging iEVs decreased splenic Th17 cells likely through M2 macrophage polarization and consequent increase in the IL1 antagonist IL1rn (Figure 3). Notably, IL1rn is a putative target of miR-125b [32]. To determine effects of miR-125b inhibition in aging iEVs on splenic Th17 cells, we examined IL17^+^ Th cells from NOD.B10.H2^b^ mice at 2 weeks after IV infusion of Ctrl or 125KD aging iEVs. In splenic CD4^+^ Th cells, 125KD but not Ctrl iEVs significantly decreased percentages of IL17^+^ cells (Figure 6A,B). Consistently, qRT-PCR assays indicated that compared with the PBS group, 125KD iEVs but not Ctrl iEVs significantly decreased the mRNA expression of Th17 markers including *IL17a*, *IL21*, and *Rorc* but increased that of Th17 inhibitor *IL1rn* in the spleen (Figure 6C). These data indicate that the transfection of miR-125b inhibitors into aging iMSCs can restore the activity of their EVs to inhibit Th17 differentiation in the spleen, likely through promoting M2 macrophage polarization.

## 3. Discussion

The immunomodulatory effects of MSC EVs and underlying mechanisms have been mainly studied using tissue-derived MSCs with high variations and limited expandability, and are far from conclusive [14,26,33,34]. Our data here indicated that at the pre-disease stage IV-infused young iEVs but not aging iEVs inhibit SS progression by directly promoting macrophage polarization toward the anti-inflammatory M2 phenotype and the consequent decrease in Th17 cells in spleen. To confirm that splenic macrophages are the key mediator of iEVs, it is necessary to determine whether the adoptive transfer of splenic macrophages isolated from NOD.B10.H2^b^ mice treated with iEVs into non-treated isogenic mice can inhibit SS progression in future studies.

Several risk factors for primary SS have been identified and a family history of autoimmune disease showed the highest odds ratio of 5.93 [35]. As indicated by clinical studies in Norway and Sweden, serum autoantibodies were present for a median 4~5 years in 66–81% of patients before the diagnosis of primary SS with high positive predictive values [36,37]. More recently, several salivary protein markers for preclinical SS have also been identified [38]. Based on screening with these risk factors and predictive markers, the early intervention of preclinical SS appears possible to prevent the sicca symptoms.

It remains unclear whether our iEVs can restore saliva secretion after the onset of primary SS, which needs be evaluated in SS mouse models at the disease stage in our future work. Both NOD and NOD.B10.H2^b^ mice show gender differences in the exocrine gland manifestations of SS with far greater immune pathology in salivary glands of females and lacrimal glands in males [25,39]. Our previous and current studies focused on effects of iEVs on the salivary gland pathology in female NOD and NOD.B10.H2^b^ mice [12,16]. Further studies in male NOD.B10.H2^b^ mice will be necessary to confirm effects of iEVs on the lacrimal gland manifestations of SS.

For the future clinical application of MSC EVs, MSCs need be expanded extensively to prepare sufficient amounts of EVs. Therefore, it is necessary to establish approaches to improve SS-inhibitory effects of aging MSC EVs. As the essential mediators of immune modulatory effects of MSC EVs [28], microRNAs are highly conserved between humans and mice [40] and can mediate cross-species communication [41]. miR-125b is highly enriched in aging iEVs compared with young iEVs [16] and reported to activate proinflammatory macrophages [29,30]. Our data confirmed that the transfection of miR-125b inhibitors into aging iMSCs restored activities of their EVs to promote M2 macrophage polarization and decrease Th17 cells in the spleen in vivo. While the direct overexpression of miR-125b in CD4^+^ T cells inhibited Th17 cell differentiation [42], we found that IV-infused iEVs were mainly taken up by splenic macrophages but not T cells, suggesting that young iEVs and 125KD aging iEVs decreased Th17 cells in the spleen indirectly through promoting M2 macrophage polarization. Moreover, the transfection of miR-125b inhibitors into aging iMSCs also increased levels of immune-suppressive miR-21 and the TGFβ1 protein in their EVs, which likely also contributed to the restoration of the SS-inhibitory effects of aging iEVs.

In conclusion, our study indicates that inhibitory effects of iEVs on SS onset are related to the increase in M2 macrophages and decrease in Th17 cells in the spleen. To maximize the production of effective iEVs from highly expanded iMSCs for future clinical application, inhibiting miR-125b in aging iMSCs appears a promising approach.

## 4. Materials and Methods

### 4.1. iMSC Culture

The human iMSCs established in our laboratory [10] were plated at a density of 500 cells per cm^2^ of growth area in complete culture medium (CCM; αMEM medium containing 17% (*v*/*v*) heat-inactivated fetal bovine serum (FBS, Atlanta Biologicals, Flowery Branch, GA, USA), penicillin-streptomycin and l-glutamine) at 37 °C and 5% CO_2_ and passaged at 70–80% confluence. To remove EVs introduced by FBS, PD 15 or PD45 iMSCs at 70–80% confluence were incubated with a serum-free and chemically defined medium (CD-CHO Medium, Invitrogen, Carlsbad, CA, USA) supplemented with HT supplements (10 mL/L; Invitrogen), 8 mM l-glutamine (Invitrogen), d-[+]-glucose (2 g/L; Sigma, St. Louis, MO, USA), 1× nonessential amino acids (Invitrogen), and 1X MEM vitamin solution (Invitrogen). After 6 h, the medium was replaced by fresh CD-CHO medium, and the conditioned medium was recovered at 48 h to isolate iMSC EVs.

### 4.2. Isolation of iMSC-EVs and Characterization

For EV isolation, the conditioned medium was filtered at 0.22 μm to remove cellular debris, and then EVs were isolated from the supernatant by ultracentrifugation at 100,000× *g* for 16 h at 4 °C using Sorvall WX Floor Ultra Centrifuge with AH-629 36 mL swinging Bucket Rotor (Thermo Fisher Scientific, Waltham, MA, USA). Isolated EVs were resuspended with PBS at concentrations of 5 to 10 × 10^10^/mL. The particle size and number of EVs were analyzed using the NanoSight LM 10 Nanoparticle Tracking Analysis System (Malvern, Malvern, UK). For in vivo biodistribution assays, iEVs were labeled with a near-infrared fluorescent dye, DiR (ThermoFisher), as reported in [17,43]. To determine types of iEV recipient cells, iEVs were labeled with a fluorescent dye, PKH26 (Sigma), as reported in [44]. Splenocytes were isolated from NOD.B10.H2^b^ mice, cultured with RMPI 1640 culture medium (Gibco, Billings, MT, USA) containing 5% FBS, treated with 3 × 10^9^ particles/mL PKH26-labeled iEVs, and then examined with flow cytometry for PKH26 signal and markers of macrophages, T cells, or B cells as detailed below.

### 4.3. Animal Studies

All animal studies were approved by the Texas A&M University (TAMU) Institutional Animal Care and Use Committee (IACUC). NOD.B10.H2^b^ mice were purchased from the Jackson Laboratory and kept in the specific pathogen-free environment maintained by TAMU Comparative Medicine Program following the NIH Guide with following room conditions: a 12 h light/12 h dark cycle, temperatures of 65–75 °F (~18–23 °C), and 40–60% humidity. Four-month-old female mice were randomly grouped for all treatments. For biodistribution assay, DiR-labeled EVs (1.5 × 10^10^ particles in 100 μL PBS) derived from young (PD15) or aging (PD45) iMSCs were injected into the tail vein. Mice were imaged with IVIS imaging system (PerkinElmer, Hopkinton, MA, USA) at 1, 3, and 24 h after iEV injection. For testing SS-inhibitory effects and mechanisms, PBS (100 μL) or EVs (1.5 × 10^10^ particles in 100 μL PBS) derived from PD15 or PD45 iMSCs were injected into the tail vein twice a week for two weeks, as we reported recently in [16]. Two weeks after the last injection, submandibular glands (SMGs) and serum were collected. The focus scores, numbers of inflammatory infiltrates of at least 50 cells per 4 mm^2^ area, were quantified from 3 H&E-stained non-consecutive sections cutting at 200 µm intervals, as recommended [45] from each of 5 SMGs per group.

### 4.4. Flow Cytometry

Spleens from NOD.B10.H2^b^ mice were minced and digested for 1 h with RPMI 1640 medium containing 1 mg/mL collagenase IV, 5 mM CaCl2, 50 mg DNase I, and 8% fetal bovine serum with continuous shaking at room temperature to prepare single cells. These cells were stained with fluorescent-labeled antibodies against F4/80 (BD Pharmingen 123116, 123107, San Diego, CA, USA), CD3 (BioLegend 100236, San Diego, CA, USA), CD19 (BioLegend 152409), CD38 (BioLegend 102707), CD206 (BioLegend 141706), CD4 (BioLegend 100408), IL17a (130-112-009, Miltenyi Biotec, San Diego, CA, USA), or the corresponding isotype controls (BioLegend 400608, 400511, 400612, 400207, 1:100). Dead cells were excluded using a LIVE/DEAD Fixable Aqua Dead Cell Stain Kit (Invitrogen, Waltham, MA, USA). The stained cells were analyzed on a CytoFlex flow cytometer (Beckman Coulter, Brea, CA, USA). Data were analyzed using the FlowJo software (Version 10.8.1, FlowJo, Ashland, OR, USA).

### 4.5. Real-Time PCR Analysis of mRNAs and miRNAs

RNAs were extracted from mouse SMGs with RNeasy Mini Kit (Qiagen, Germantown, MD, USA) and reverse transcribed with High-Capacity cDNA Reverse Transcription Kit (Applied Biosystems, Waltham, MA, USA). The qPCR was performed with SYBR Green Master Mix (Bio-Rad, Hercules, CA, USA) on a CFX Connect PCR System (Bio-Rad). The primers were synthesized by Invitrogen with sequences retrieved from Primerbank (http://pga.mgh.harvard.edu/primerbank, accessed on 1 June 2021). The qPCR data were analyzed with Gapdh as the reference gene. Total RNA was isolated from ultracentrifuged EVs (1 × 10^11^ particles) with the EZNA Total RNA Kit (Omega Bio-Tek, Doraville, CA, USA). Levels of miR-21 and miR-125b were measured with corresponding TaqMan MicroRNA Assay kits and normalized by miR-143 that was consistently expressed in young and aging EVs [16]. N = 3 for PCR analyses of iEVs and 5 for mouse samples.

### 4.6. Cell Transfection

Cells with 60% confluence were transfected with 20 nM miRNA inhibitors or mimics for control or miR-125b (Invitrogen) using Lipofectamine RNAiMAX (Invitrogen) for 5 h. After transfection, cells were recovered with antibiotic-free CCM overnight for collecting EVs.

### 4.7. ELISA

Human TGFβ1 in iEVs (1 × 10^10^ particle/mL) and Anti-La in serum from NOD.B10.H2^b^ mice were measured by commercial ELISA kits (R&D Systems, Minneapolis, MN, USA; Signosis, Santa Clara, CA, USA) according to the manufacturer’s protocol.

### 4.8. Statistics

Column data with one grouping variable were analyzed using one-way ANOVA, and grouped data with two grouping variables were analyzed using two-way ANOVA, both followed by Tukey’s multiple comparison tests. Statistical analysis and graphical generation of data were conducted with GraphPad Prism 9 software Version 9.4.1 (San Diego, CA, USA).

## Data Availability

Not applicable.

## Supplementary Materials

The following supporting information can be downloaded at: https://www.mdpi.com/article/10.3390/ijms24065258/s1.
