# Supplementary material for: Inhibitory Effects of Extracellular Vesicles from iPS-Cell-Derived Mesenchymal Stem Cells on the Onset of Sialadenitis in Sjögren’s Syndrome Are Mediated by Immunomodulatory Splenocytes and Improved by Inhibiting miR-125b
Source: Int J Mol Sci. 2023 Mar 9;24(6):5258. doi: 10.3390/ijms24065258 (PMC10049013; doi:10.3390/ijms24065258)
Supplement: Supplementary file 1 [file ijms-24-05258-s001.zip › ijms-2212130-supplementary.pdf]

Supplementary information

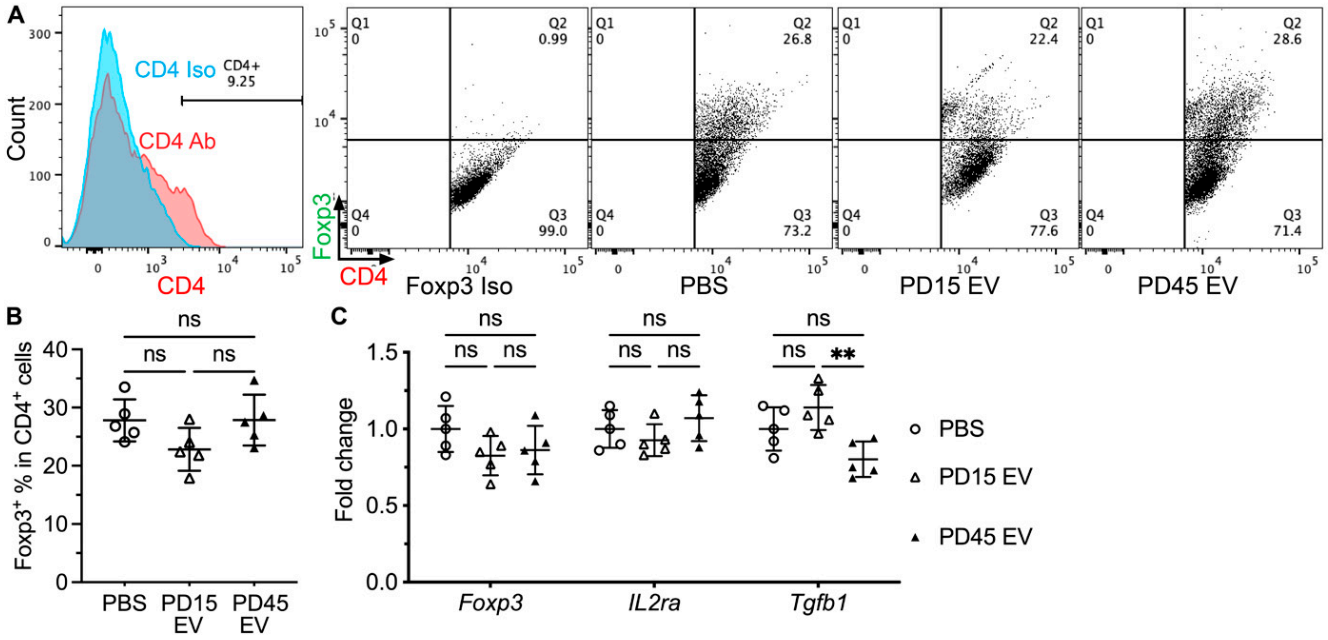

Figure S1. Effects iEVs on splenic Treg cells.

Four-month-old female NOD.B10.H2^{b} mice were IV injected with PBS, PD15 iEVs, or PD45 iEVs twice a week for two weeks. Splenocytes were collected at two weeks after last injection.

A,B: Percentages of Foxp3^{+} cells in CD4^{+} splenocytes were examined with flow cytometry.

C: The mRNA levels of markers for Treg cells in spleen were examined with qRT-PCR.

N = 5. ns: not significant. **: $p < 0.01$.
